# Diagnostic yield of endoscopy in patients with abdominal complaints: incremental value of faecal calprotectin on guidelines of appropriateness

**DOI:** 10.1186/1471-230X-14-57

**Published:** 2014-03-29

**Authors:** Emanuel Burri, Michael Manz, Patricia Schroeder, Florian Froehlich, Livio Rossi, Christoph Beglinger, Frank Serge Lehmann

**Affiliations:** 1 Department of Gastroenterology & Hepatology, University Hospital Basel, Petersgraben 4, 4031 Basel, Switzerland; 2 Division of Gastroenterology, Department of Internal Medicine, Cantonal Hospital Liestal, Rheinstrasse 26, 4410 Liestal, Switzerland; 3 Department of Gastroenterology, St. Claraspital, Kleinriehenstrasse 30, 4058 Basel, Switzerland

**Keywords:** Esophagogastroduodenoscopy, Colonoscopy, Appropriateness, Calprotectin, Diagnostic accuracy

## Abstract

**Background:**

European Panel on the Appropriateness of Gastrointestinal Endoscopy (EPAGE) criteria have been developed to increase diagnostic yield, but their predictive value is limited. We investigated the incremental diagnostic value of faecal calprotectin to EPAGE criteria.

**Methods:**

In a post-hoc analysis of a prospective study, EPAGE criteria were applied to 298 of 575 (51.8%) patients who had undergone esophagogastroduodenoscopy (EGD), colonoscopy or both for abdominal complaints at the Division of Gastroenterology & Hepatology at the University Hospital Basel in Switzerland. Faecal calprotectin was measured in stool samples collected within 24 hours before the investigation using an enzyme-linked immunosorbent assay. Final endoscopic diagnoses were blinded to calprotectin values.

**Results:**

Of 149 EGDs and 224 colonoscopies, 17.6% and 14.7% respectively were judged inappropriate by EPAGE criteria. Appropriate or uncertain indications revealed more endoscopic findings in both EGD (46.3% vs. 23.1%, P = 0.049) and colonoscopy (23.6% vs. 6.1%, P = 0.041) than inappropriate indications. Median calprotectin levels were higher (81.5 μg/g, interquartile range 26-175, vs. 10 μg/g, IQR 10–22, P < 0.001) and testing was more often positive (>50 μg/g) in patients with endoscopic findings, both in EGD (58.2% vs. 33.0%, P = 0.005) and in colonoscopy (57.3% vs. 7.4%, P < 0.001). The use of faecal calprotectin in addition to EPAGE criteria improved the risk reclassification of patients by endoscopic findings. The calculated net reclassification index was 37.8% (P = 0.002) for EGD and 110.9% (P <0.001) for colonoscopy, thus improving diagnostic yield to 56.8% and 70.2%, respectively.

**Conclusions:**

The use of faecal calprotectin in addition to EPAGE criteria improved diagnostic yield in patients with abdominal complaints.

## Background

Abdominal complaints are commonly seen in patients in clinical practice and many undergo endoscopy for further evaluation. However, symptoms may arise from a variety of disorders, including functional dyspepsia and irritable bowel syndrome, and the potential risk of invasive procedures must be balanced against the benefit of detecting a significant organic disease.

Patient selection based on symptoms alone is unfortunately not reliable, both for patients with dyspepsia [1] and with lower abdominal symptoms [2]. Around half of patients with peptic ulcer disease or esophagitis at endoscopy will be misclassified when presenting with epigastric pain [1]. Accordingly, major pathologies (ulcer, malignancy) are found in only a minority of dyspeptic patients [3]. Similarly, in average-risk patients with non-specific lower abdominal symptoms, the overall yield of colonoscopy is low [2] and may be similar to an average-risk screening population. For patients reporting hematochezia, 66% of patients <45 years will have normal findings and only 17% will show significant lesions [4]. There is clearly a need for better selection criteria to decide on endoscopy and improve the diagnostic yield in patients with abdominal complaints.

The *European Panel on the Appropriateness of Gastrointestinal Endoscopy* (EPAGE) has published a series of guidelines on the appropriate use of endoscopy for a variety of clinical scenarios (http://www.epage.ch) [5,6]. Using these criteria, inappropriate indications for esophagogastroduodenoscopy (EGD) or colonoscopy have been reported in 10.5 – 39% [7-18], depending on patient selection. Accordingly, EGD judged appropriate or uncertain by EPAGE guidelines yielded significantly more relevant lesions (60%) than did those judged to be inappropriate (37%) [7]. These findings were confirmed by some studies [15,16] but not by all [19]. In patients undergoing screening colonoscopy, 14.4% had significant findings and compared to inappropriate indications, the odds ratio of endoscopic findings for appropriate or uncertain indications was 3.2 (95%CI 1.1-17) [10]. Similar results have been reported in consecutive patients referred for diagnostic colonoscopy [9,11,12,17,19]. The efficient use of endoscopic procedures is paramount to ensure high-quality cost-effective medical care. However, the low specificity of current guidelines of appropriateness substantially reduces the predictive value of relevant endoscopic findings. The use of a diagnostic test in addition to appropriateness criteria might therefore be beneficial by increasing diagnostic yield.

Calprotectin is an abundant, calcium- and zinc-binding protein found mainly in neutrophils. It correlates well with neutrophil infiltration of the intestinal mucosa and when measured in faeces, it is considered as an established biological marker of intestinal inflammation throughout the gastrointestinal tract. It has proven highly useful for the identification of inflammatory bowel disease [20] and for distinguishing between organic and functional disorders of the colon [21] and similar the upper intestinal tract although less performant [22].

The goal of this study was thus to investigate if the use of faecal calprotectin testing in combination with guidelines of appropriateness would improve the diagnostic yield of endoscopic procedures. To do so, we investigated a large population of unselected patients with abdominal discomfort referred for endoscopy.

## Methods

### Setting and participants

We performed a post-hoc analysis of a prospective study to investigate the value of guidelines of appropriateness and faecal calprotectin levels on diagnostic yield in patients undergoing gastrointestinal endoscopy for abdominal discomfort [22]. The study was conducted at the Division of Gastroenterology & Hepatology of the University Hospital Basel in Switzerland. A total of 575 patients with abdominal discomfort referred for either EGD or colonoscopy were enrolled in the study. Patients <18 years old were excluded. The study was carried out according to the principles of the Declaration of Helsinki and the local ethics committees (EKBB – Ethikkommission beider Basel, Switzerland) approved the protocol. All patients provided written informed consent before participating in any protocol-specific procedures.

### Adjudication of the final diagnosis

The final diagnosis was independently adjudicated by two gastroenterologists not involved in clinical of study patients and blinded to calprotectin test results on the basis of all available medical records pertaining to the individual patient (clinical data, laboratory values, endoscopy report, histology report) according to current recommendations. Senior gastroenterologists who were unaware of faecal test results performed all endoscopies and findings were documented on a computer-based datasheet (ViewPoint, GE Healthcare, Chalfont St Giles, U.K.).

### Endpoint

The diagnostic value of calprotectin measurement and guidelines of appropriateness were assessed in comparison to the presence of clinically significant findings at endoscopy.

### Assessing appropriateness of endoscopy

We used the *European Panel on the Appropriateness of Gastrointestinal Endoscopy (EPAGE)* criteria to assess the appropriateness of endoscopy. Briefly, members of a multidisciplinary European expert panel examined existing evidence summarized in a comprehensive literature review and rated the appropriateness of all possible indications for endoscopy in a series of clinical indications. In each clinical scenario, the use of endoscopy was then graded on a 9-point scale using the following scores: 1 = extremely inappropriate, 5 = uncertain, 9 = extremely appropriate. An indication for endoscopy was considered appropriate if the median rating was between 7 and 9, without disagreement, and inappropriate if the median was between 1 and 3, without disagreement. Scenarios with a median rating of 4 to 6, or those revealing disagreement among the panellists were considered “uncertain” as to the appropriateness of endoscopy in such cases. A more detailed explanation of the methodology and appropriateness ratings can be found elsewhere [5,6].

In our study, we retrospectively assessed EPAGE criteria for endoscopy in our population of patients with abdominal discomfort. Using an openly-accessible computer-based algorithm, the appropriateness of endoscopy was determined by two gastroenterologists blinded to FC values. In case of disagreement, cases were reviewed in conjunction with a third gastroenterologist who was considered to be an expert in the field.

Thirty-four groups of indications for EGD and colonoscopy have been described in the EPAGE criteria. In our study, patients were categorized in a predefined set of clinical scenarios, according to their main symptoms at presentation. If patients did not fit any of these criteria or if insufficient data was available from chart review to determine EPAGE criteria, patients were excluded from the final analysis.

### Collection of faecal samples

All tests were performed on a single stool sample in all patients. Patients were instructed to collect the sample at home 24 hours prior to bowel preparation or endoscopy. Samples were delivered on the day of the investigation and stored in a refrigerator before transfer to the study laboratory (Rothen Medical Laboratories, Basel, Switzerland) within 48 hours for analysis. Calprotectin is stable at room temperature for up to seven days. All faecal samples were processed within 72 hours after collection. The laboratory personnel carrying out the analysis was blinded to the clinical history, clinical data and the endoscopic findings of the patients.

### Measurement of faecal calprotectin

Faecal calprotectin values were determined using a commercially-available enzyme-linked immunosorbent assay (ELISA) by Bühlmann Laboratories AG, Schönenbuch, Switzerland. Senior laboratory personnel blinded to patient history and calprotectin levels performed all analyses.

Aliquots of approximately 100 mg of faeces were homogenized in a 5 mL extraction buffer delivered with the assay. 2 mL of the homogenate was then centrifuged in a micro-centrifuge for 5 min at 3000 g and 100 μl of the diluted supernatant (1:50 with incubation buffer) were incubated at room temperature onto a microtiter plate coated with a monoclonal capture antibody highly specific to the calprotectin heterodimeric and polymeric complexes. After incubation, washing and the addition of a detection antibody coupled to horseradish peroxidase, substrate was added and incubated, followed by addition of a stop solution. The absorption rate was determined at an optical density of 450 nm. The test measured concentrations from 10 to 600 μg calprotectin/g feces with an intra- and inter-assay coefficient of 4.7% and 4.1%, respectively. For quantification of higher calprotectin concentrations, additional dilutions of the extracts were done. The cut-off level representing a positive value was >50ug/g as recommended by the manufacturer.

### Endoscopy

All patients underwent standard endoscopy performed by senior gastroenterologists who were unaware of faecal calprotectin values at the time of the investigation. Endoscopies were documented on a computer-based datasheet (ViewPoint, GE Healthcare, Chalfont St. Giles, U.K.) that included a detailed description of the findings by choosing from a predefined list and electronic storage of all images taken during the investigation. Biopsies were collected if appropriate as decided by the endoscopist. Patients with no significant lesion but with elevated faecal calprotectin levels (> 50 μg/g) at initial endoscopy were further investigated with either EGD or colonoscopy. The endoscopist performing the follow-up endoscopy was aware of the indication for the investigation (positive test result).

### Statistical analysis

Results of numerical data are presented as mean (standard deviation, SD) or median (interquartile range, IQR) where appropriate. The Mann–Whitney *U*-test (for two independent groups) and the Kruskal-Wallis H-test (for more than two independent groups) were used to compare numerical data and the chi-square test was used to compare categorical data. Receiver operating characteristics analyses were carried out to determine the test characteristics. Overall accuracy was calculated according to the following formula: (true positive test results + true negative test results)/total population. Improvements in risk classification for significant findings were evaluated using the net reclassification improvement (NRI) method. Predicted probabilities for significant findings on endoscopy for each patient were determined using the base model (EPAGE scoring). The NRI represents the percentage change in predicted findings after including a new marker in the base model. Owing to the lack of established risk prediction models and given an observed prevalence for significant findings at endoscopy of 36%, risk categories of <30%, 30 – 50% and >50% were chosen for inappropriate, uncertain and appropriate endoscopy from a clinical point of view. Model 1 included scoring from EPAGE criteria; the new model 2 included faecal calprotectin in addition to the EPAGE score as a continuous parameter. An additive logistic regression model was performed. The predicted values of the regression were considered as a risk factor. Before evaluation, faecal calprotectin values were log-transformed. We then categorized patients according to their risk of significant endoscopic findings and compared the proportion of patients whose new prediction was improved with those whose prediction became less accurate using the new model 2. A related parameter, the integrated discrimination improvement (IDI), represents the increase in discriminatory power of the model with an additional marker by comparing average predicted probabilities between patients with and without significant findings of the two models without first categorizing the probabilities. A p-value <0.05 was considered to be statistically significant. To visualize the estimate risks depending on the predictors, nomograms were displayed. All evaluations were done using R version 2.15.1 (R Core Team (2012), Vienna, Austria. ISBN 3-900051-07-0, URL http://www.R-project.org/) and MedCalc version 12.2.1 (MedCalc Software, Mariakerke, Belgium).

## Results

### Patients characteristics

**Figure 1:**
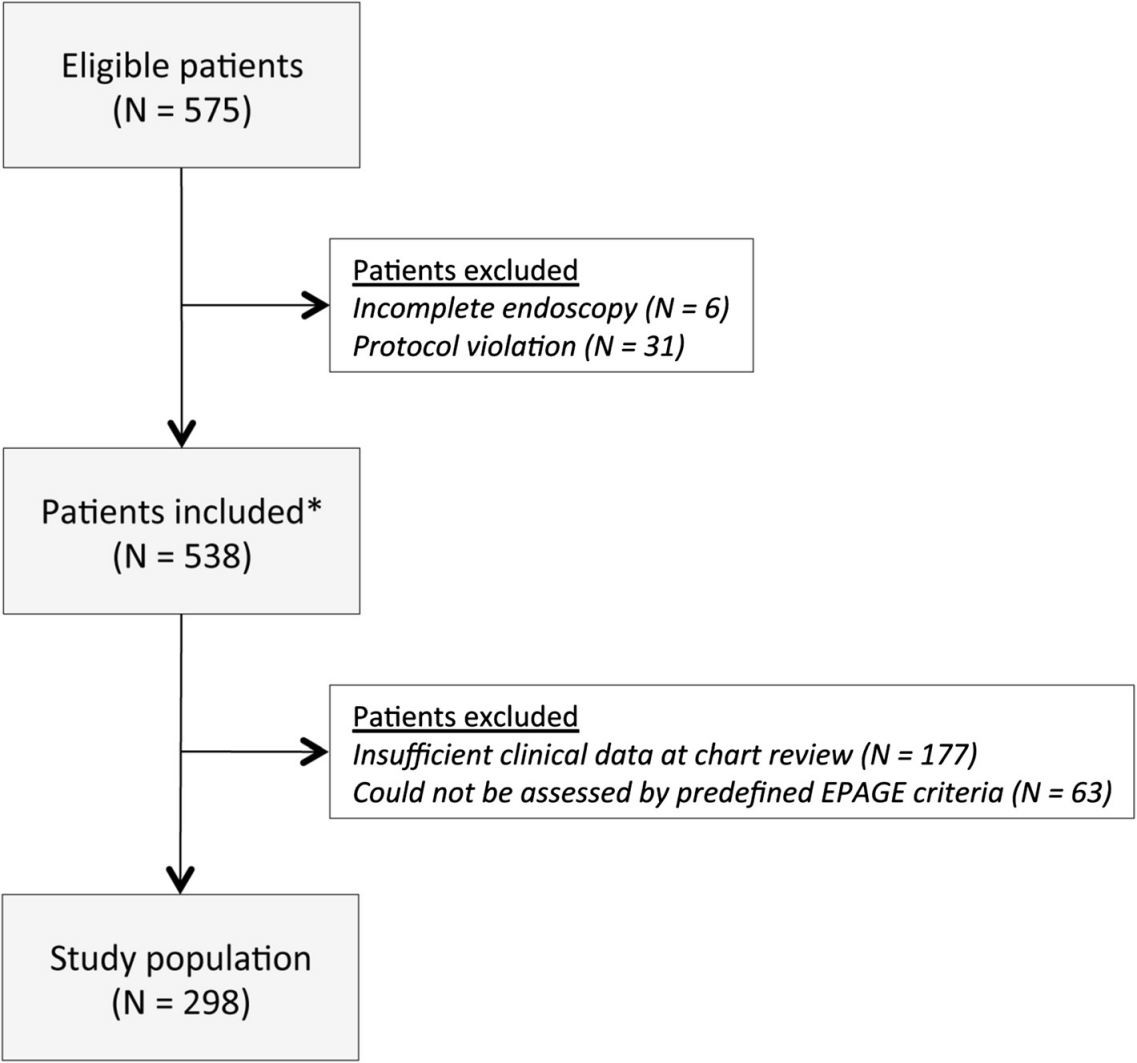
**Study flow.** Study flow of patients referred for endoscopy. A total of 277 patients were excluded: 6 patients because of incomplete endoscopy, 31 patients because of protocol violation (no follow-up investigation in normal endoscopy but faecal calprotectin > 50 μg/g), 244 patients because of insufficient data at chart review to calculate EPAGE score and 63 patients because they could not be assessed using the predefined list of EPAGE criteria. * Patients included in [22].

Of 575 patients enrolled in the study, 298 patients (51.8%) were available for analysis. Two hundred and seventy-seven patients (48.2%) were excluded; in 177 patients data from chart review was insufficient to calculate an EPAGE score, 63 patients could not be adjudicated according to our predefined list EPAGE algorithms and 37 patients were excluded for protocol violation (did not have follow-up investigation after negative endoscopy and positive calprotectin test result) or incomplete endoscopy (Figure 1). Baseline characteristics are shown in Table 1. EGD was performed in 149 patients (50.0%) and 224 (75.2%) had colonoscopy. In 75 patients (25.2%) both procedures were performed. Among the study population, the overall prevalence of a significant lesion in the gastrointestinal tract was 36.2%, specifically 42.3% in the upper gastrointestinal tract and 21.0% in the colon.

**Table 1:** Baseline characteristics

Number of patients	298
Female patients, N (%)	164 (55.0%)
Age, years (IQR)	58 (46 – 67)
Colonoscopy, N (%)	224 (75.2%)
EGD, N (%)	149 (50.0%)
Colonoscopy and EGD, N (%)	75 (25.2%)

Data are presented as median (interquartile range, IQR) and number of patients (%). EGD: esophagogastroduodenoscopy.

### Appropriateness of endoscopy

Overall, 373 endoscopies were performed. Among the predefined list of EPAGE indications, the investigation of uncomplicated dyspepsia (N = 67, 44.9%) was the most prevalent reason for performing EGD, while colonoscopy was most often done for lower abdominal symptoms (N = 114, 50.9%) (Table 2). Of 149 EGDs performed, 26 (17.4%) were for inappropriate indications, whereas 10 (6.7%) were for indications that were considered uncertain and 113 (75.8%) were for appropriate indications. Similarly, of 224 colonoscopies performed, 33 (14.7%) were considered inappropriate, 51 (22.8%) were judged uncertain and 140 (62.5%) were done for appropriate indications. Table 2 gives a detailed overview. The investigation of uncomplicated dyspepsia showed the highest rate of inappropriate indications (29.9%), whereas the investigation of alarm symptoms was done for an appropriate indication in a majority of patients (97.8%). Overall, the rate of inappropriate indications for endoscopy was 15.8%.

**Table 2:** Indication and appropriateness of endoscopy

**Indication**	**N**	**Inappropriate**	**Uncertain**	**Appropriate**
**EGD**	**149**	**26 (17.5%)**	**10 (6.7%)**	**113 (75.8%)**
Uncomplicated dyspepsia	67	20 (29.9%)	7 (10.4%)	40 (59.7%)
Frequent symptoms suggesting reflux disease	27	5 (18.5%)	2 (7.4%)	20 (74.1%)
Atypical chest pain	1	1 (100.0%)	0 (0.0%)	0 (0.0%)
Alarm symptoms	46	0 (0.0%)	1 (2.2%)	45 (97.8%)
Miscellaneous	8	0 (0.0%)	0 (0.0%)	8 (100%)
**Colonoscopy**	**224**	**33 (14.7%)**	**51 (22.8%)**	**140 (62.5%)**
Iron-deficiency anemia	25	2 (8.0%)	8 (32.0%)	15 (60.0%)
Hematochezia	61	1 (1.6%)	7 (11.5%)	53 (86.9%)
Lower abdominal symptoms	114	28 (24.6%)	36 (31.6%)	50 (43.8%)
Uncomplicated diarrhea	15	1 (6.7%)	0 (0.0%)	14 (93.3%)
Miscellaneous	9	1 (11.1%)	0 (0.0%)	8 (88.9%)
**Total**	**373**	**59 (15.8%)**	**61 (16.4%)**	**253 (67.8%)**

Data are presented as numbers (%). Miscellaneous indications for EGD included 8 patients with celiac disease; miscellaneous indications for colonoscopy included 6 patients with metastases that required further evaluation, 2 patients with iron-deficiency without anaemia and 1 patient with perianal fistula.

### Diagnostic yield in inappropriate, uncertain and appropriate endoscopy

Using EPAGE criteria, diagnostic yield for clinically significant lesions was higher in patients with appropriate or uncertain indications than in those with an inappropriate indication for endoscopy (32.5% vs. 13.6%, P = 0.006). EGD revealed significant findings in 46.3% of appropriate or uncertain indications compared to 23.1% in inappropriate indications (P = 0.049). Similarly, significant findings during colonoscopy were more prevalent in patients with appropriate or uncertain indications (23.6%) compared to inappropriate indications (6.1%, P = 0.041). Endoscopic findings and corresponding appropriateness ratings are given in Table 3.

**Table 3:** Diagnostic yield of endoscopy

**Endoscopic findings**	**N**	**Inappropriate**	**Uncertain**	**Appropriate**
**No significant findings**	**261**	**51 (19.6%)**	**45 (17.2%)**	**165 (63.2%)**
**Clinically-significant findings**	**112**	**8 (7.1%)**	**16 (14.3%)**	**88 (78.6%)**
*Reflux esophagitis*	*44*	*5 (11.3%)*	*2 (4.6%)*	*37 (84.1%)*
*Erosive gastritis*	*13*	*1 (7.7%)*	*1 (7.7%)*	*11 (84.6%)*
*Gastric ulcer*	*4*	*0 (0.0%)*	*0 (0.0%)*	*4 (100%)*
*Stomach cancer*	*2*	*0 (0.0%)*	*1 (50%)*	*1 (50%)*
*Inflammatory bowel disease*	*9*	*0 (0.0%)*	*4 (44.4%)*	*5 (55.6%)*
*Infectious colitis*	*4*	*0 (0.0%)*	*0 (0.0%)*	*4 (100%)*
*Adenomatous polyp*	*20*	*1 (5.0%)*	*4 (20.0%)*	*15 (75.0%)*
*Colonic cancer*	*10*	*0 (0.0%)*	*2 (20.0%)*	*8 (80.0%)*
*Miscellaneous*	*6*	*1 (16.7%)*	*2 (33.3%)*	*3 (50.0%)*

Data are presented as numbers (%). Reflux esophagitis included Los Angeles grade A (N = 22), grade B (N = 11), grade C (N = 3) and grade D (N = 8). Inflammatory bowel disease included Crohn’s disease (N = 4) and ulcerative colitis (N = 5). Miscellaneous included diverticulitis (N = 2), NSAID-induced colitis (N = 2) and unspecified proctitis (N = 2).

### Diagnostic value of faecal calprotectin

**Figure 2:**
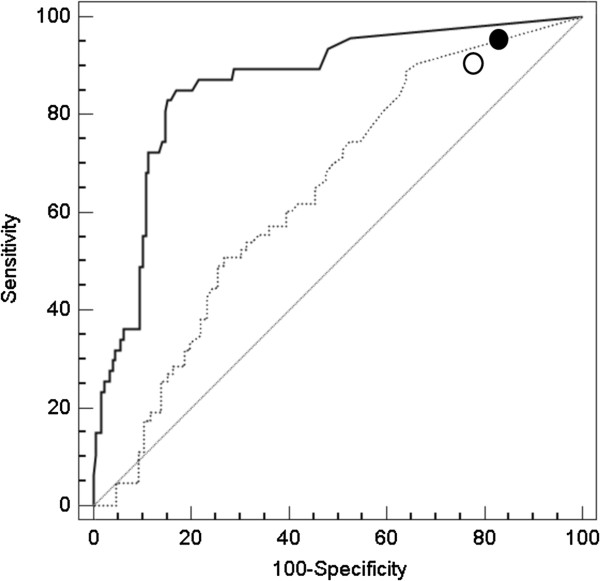
**Diagnostic performance of faecal calprotectin testing.** Receiver-operating characteristic (ROC) curve for clinically significant findings during EGD (dotted line) and colonoscopy (solid line). We also report the accuracy values for EPAGE criteria (EGD: open circle, colonoscopy: solid circle). The ROC curve represents the relationship between sensitivity and specificity for a considered outcome. EPAGE, European Panel on the Appropriateness of Gastrointestinal Endoscopy.

Median calprotectin levels were higher in patients with significant findings (N = 108, median 81.5 μg/g, IQR 26 – 175 μg/g) than in patients without such findings (N = 190, 10 μg/g, IQR 10 – 22, P < 0.001). Using evaluation of faecal calprotectin as a diagnostic test, we found an area under the receiver operating characteristics curve (AUC) of 0.846 (95% confidence interval (CI), 0.80 – 0.89), specifically an AUC of 0.642 (0.56 – 0.72) for EGD and an AUC of 0.863 (0.81 – 0.91) for colonoscopy (Figure 2). Using the optimal cut-off value (58 μg/g for EGD and 38 μg/g for colonoscopy), faecal calprotectin yielded a sensitivity and specificity of 49.2% and 74.4% for EGD and 72.3% and 88.7% for colonoscopy, respectively. Thus, patients with positive calprotectin test results (> 50 μg/g according to the manufacturer) more often had significant findings at endoscopy, both at EGD (58.2% vs. 34.1%, P = 0.005) and at colonoscopy (57.4% vs. 7.4%, P < 0.001).

### Incremental value of calprotectin to EPAGE criteria

**Figure 3:**
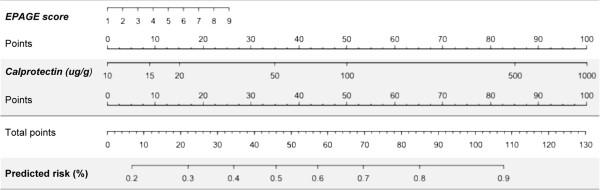
**Nomogram for risk prediction of clinically significant findings by means of EPAGE criteria and faecal calprotectin values for EGD.** Nomograms of EPAGE scoring and log^10^ faecal calprotectin values to predict the risk of significant findings at EGD. Points for EPAGE scoring and faecal calprotectin values (ug/g) are added to a total score, which will indicate the predicted risk (%) for endoscopic findings.

**Figure 4:**
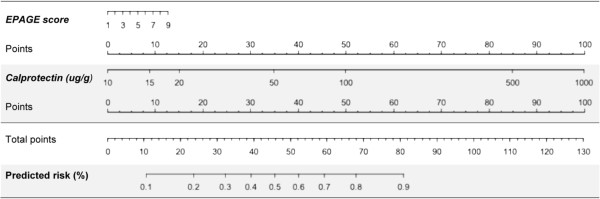
**Nomogram for risk prediction of clinically significant findings by means of EPAGE criteria and faecal calprotectin values for colonoscopy.** Nomograms of EPAGE scoring and log^10^ faecal calprotectin values to predict the risk of significant findings at colonoscopy. Points for EPAGE scoring and faecal calprotectin values (ug/g) are added to a total score, which will indicate the predicted risk (%) for endoscopic findings.

The use of faecal calprotectin in addition to EPAGE criteria improved the reclassification of patients in terms of risk of significant findings at endoscopy (Tables 4 and 5). Amongst patients with normal EGD, 46 patients (63%) were correctly reclassified as at lower risk of endoscopic findings and 4 patients (5.5%) were incorrectly reclassified as at higher risk. Amongst patients with significant findings at EGD, 11 patients (14.5%) were correctly reclassified as at higher risk of endoscopic findings and 26 patients (34.2%) were incorrectly reclassified as at lower risk. The calculated net reclassification index (NRI) was 37.8% (P 0.002) and absolute integrated discrimination index (IDI) was 0.18 (P < 0.001). For colonoscopy, 100 patients (66.7%) with normal findings were correctly reclassified as at lower risk for endoscopic lesions and 12 patients (8.0%) were incorrectly reclassified as at higher risk. Amongst patients with significant findings, 49 patients (66.2%) were correctly reclassified as at higher risk for endoscopic findings and 11 patients (14.9%) were incorrectly reclassified as at lower risk. The calculated NRI was 110.9% (P <0.001) and the absolute IDI was 0.41 (P <0.001). Figures 3 and 4 show nomograms of EPAGE scoring and faecal calprotectin values to predict the risk of significant findings at endoscopy. Using EPAGE criteria in conjunction with faecal calprotectin testing increased diagnostic yield (56.8% for EGD; 70.2% for colonoscopy) compared to EPAGE criteria (46.3% and 23.6%, respectively), and measurement of faecal calprotectin alone, especially for colonoscopy (58.2% and 57.4%, respectively).

**Table 4:** Reclassification of patients with normal and clinically significant findings undergoing EGD by means of faecal calprotectin measurement

**EGD**	**Endoscopic finding probability model 2 including EPAGE criteria and faecal calprotectin values**				
	**Probability of findings**	**<30%**	**30-50%**	**>50%**	**Total**
Endoscopic finding probability model 1 including EPAGE criteria	*No findings*				
	<30%	0	0	0	0
	30-50%	13	6	4	23
	>50%	6	27	17	50
	Total	19	33	21	73
	*Significant findings*				
	<30%	0	0	0	0
	30-50%	3	0	11	14
	>50%	2	21	39	62
	Total	5	21	50	76

Amongst patients with normal EGD, 46 patients (63.0%) were correctly reclassified as at lower risk of endoscopic findings and 4 patients (5.5%) were incorrectly reclassified as at higher risk. Amongst patients with significant findings at EGD, 11 patients (14.5%) were correctly reclassified as at higher risk of significant findings and 26 patients (34.2%) were incorrectly reclassified as at lower risk. The calculated net reclassification index (NRI) was 37.8% (P 0.002).

**Table 5:** Reclassification of patients with normal and clinically significant findings undergoing colonoscopy by means of faecal calprotectin measurement

**Colonoscopy**	**Endoscopic findings probability model 2 including EPAGE criteria and faecal calprotectin values**				
	**Probability of findings**	**<30%**	**30-50%**	**>50%**	**Total**
Endoscopic finding probability model 1 including EPAGE criteria	*No findings*				
	<30%	25	1	1	27
	30-50%	100	13	10	123
	>50%	0	0	0	0
	Total	125	14	11	150
	*Significant findings*				
	<30%	4	0	6	10
	30-50%	11	10	43	64
	>50%	0	0	0	0
	Total	15	10	49	74

Amongst patients with normal colonoscopy, 100 patients (66.7%) were correctly reclassified as at lower risk for endoscopic lesions and 12 patients (8.0%) were incorrectly reclassified as at higher risk. Amongst patients with significant findings at colonoscopy, 49 patients (66.2%) were correctly reclassified as at higher risk of endoscopic findings and 11 patients (14.9%) were incorrectly reclassified as at lower risk. The calculated NRI was 110.9% (P <0.001).

## Discussion

This post-hoc analysis of a prospective cohort of patients with abdominal discomfort undergoing upper and lower GI endoscopy examined the value of the European Panel on the Appropriateness of Gastrointestinal Endoscopy (EPAGE) criteria and faecal calprotectin testing on diagnostic yield. Specifically, we investigated if the addition of faecal calprotectin testing would decrease the rate of inappropriate endoscopies and increase diagnostic yield. We report the following findings: First, the rate of inappropriate endoscopies in our study was slightly lower than in most studies reported in the literature, owing to the fact that only symptomatic patients were included. Second, the diagnostic yield of clinically significant findings was higher for appropriate than for inappropriate endoscopies, both for EGD and colonoscopy, confirming prior findings [7,9,11,12,15-18]. Third, faecal calprotectin testing provided a significant added value to the ability to detect significant findings throughout the gut while performing less accurately in the upper intestinal tract. Accordingly, diagnostic yield was significantly higher in patients with elevated calprotectin values. Fourth, faecal calprotectin testing improved risk stratification in patients with abdominal complaints when combined with EPAGE criteria. The NRI was 37.8% and 110.9% and the absolute IDI was 0.18 and 0.41 for patients undergoing EGD and colonoscopy, respectively. Therefore, our data support the use of faecal calprotectin testing in conjunction with EPAGE criteria in selecting the right patients for endoscopy. The integration of calprotectin values in appropriateness algorithms would likely increase diagnostic yield of endoscopic investigations.

The number of endoscopic procedures, especially colonoscopies, performed is steadily increasing, both in Europe and the USA [23,24]. In the light of limited resources and ever-increasing health-care costs, optimizing the appropriate selection of patients for endoscopy is crucial. Unfortunately, patient selection based on symptoms alone is not suitable [1,2,25] and physicians traditionally rely on clinical signs, laboratory data, expert knowledge of the literature and personal experience to decide whether endoscopy has to be performed.

In an effort to improve the appropriate use of endoscopy and ultimately increase diagnostic yield, the multidisciplinary European Panel on the Appropriateness of Gastrointestinal Endoscopy (EPAGE) has developed guidelines for a series of clinical scenarios [5,6]. Inappropriate EGDs and colonoscopies are associated with lower diagnostic yield, procedural risks for no apparent health benefit and longer waiting periods in endoscopy units. The rate of inappropriate endoscopies in our study population was 15.8%, specifically 17.4% for EGD and 14.7% for colonoscopy. This was slightly lower than inappropriate rates reported by most studies ranging from 19.5% to 39% [7,8] for EGD and 10.5% to 27% for colonoscopy [9-18]. However, the type of criteria used and patients included may render direct comparison of inappropriateness rates difficult. In our study which included only symptomatic patients, inappropriate endoscopies were most often done for uncomplicated dyspepsia (29.9%) and lower abdominal symptoms (24.6%).

By linking clinical indications and endoscopic findings, diagnostic yield can be determined and the prevalence of endoscopic findings may differ significantly depending on the indication. For example, colonoscopy for gastrointestinal bleeding (haematochezia, iron-deficiency anaemia, melena) will detect more colorectal cancers (1 in 9 to 13 endoscopies) than in patients without signs of bleeding (1 in 109 colonoscopies) [26]. Similarly, diagnostic yield of significant findings in screening patients is low (14.4%) [10]. Available data on the prevalence of significant findings in patients referred for diagnostic endoscopy in an open-access system such as that which exists in Switzerland [7,10-12,15,17] show the following: EGD judged appropriate or uncertain yielded significantly more relevant lesions (60%) than those judged inappropriate (37%) [7]. In patients referred for colonoscopy, appropriate or uncertain indications had more relevant endoscopic findings than those with inappropriate indications (42% vs. 21% [11], 38.8% vs. 24.5% [12], 39.2% vs 13.4% [13], 74% vs. 16% [15], 25.6% vs. 17.6% [17]) and adherence to EPAGE recommendations was an independent predictor of significant findings (OR 1.93) [12]. In addition, alarm symptoms are not effective predictors of endoscopic findings [27-29]. Major pathologies were found in only 21% (787 of 3815) of upper endoscopies [3] and 42% of EGDs performed for severe alarm symptoms such as dysphagia or hematemesis did not result in pathological findings [30]. In a model incorporating three Manning criteria and alarm features yielded a correct diagnosis of IBS in 96% and a correct diagnosis of organic disease in 52% of cases [29]. Similarly, alarm features did not discriminate functional dyspepsia from upper gastrointestinal disease. Vakil et al. showed in their systematic review and meta-analysis, that alarm symptoms have a low positive predictive value for malignancy at EGD [27]. If alarm symptoms are present, the likelihood of an upper GI malignancy increases slightly, but the absolute increase remains small. The negative predictive will be high, but this reflects the low prevalence of cancer in dyspeptic patients. In patients with constipation, the risk of a significant finding at colonoscopy was not increased and if present alone, the risk was lower than for average-risk screening colonoscopy [31]. Even when haematochezia was present, colonoscopy in patients with a “low and average risk” for colorectal cancer showed abnormal findings in only 34% of younger (age <45 years) compared to 66% in older patients [4].

Data from our study confirms previous results by showing a higher diagnostic yield in appropriate compared to inappropriate investigations both for EGD and colonoscopy. Also, the overall prevalence of clinically-significant findings in our study population was 36.2% (EGD 43.2%, colonoscopy 21.0%) and comparable to findings by others for EGD (30 – 52%) [7,8,15,30] and colonoscopy (14.4 – 41%) [10-13,17,18].

Unfortunately, the association between appropriateness criteria and the detection of significant lesions is less than perfect such as is the relationship between clinical symptoms and endoscopic diagnosis, even in patients with clinical signs suggesting organic disease, e.g. patients with haematochezia, chronic diarrhoea or constipation. Relevant endoscopic findings are detected in a considerable proportion of inappropriate EGDs and colonoscopies [7,8] and diagnostic yield in appropriate endoscopies is <50% in most studies. As a result, nearly one in every two patients with an appropriate indication will have a normal examination [32]. Improving diagnostic yield of endoscopies is therefore highly desirable.

In our study, we measured faecal calprotectin as an additional biomarker to improve diagnostic yield in symptomatic patients referred for endoscopy in an open-access system. Patients with endoscopic findings more often had positive faecal calprotectin testing than patients with normal findings and as a stand-alone test, it was useful in identifying organic intestinal disease, especially in the colon. It has been shown, that the diagnostic value of fecal calprotectin is not limited to the colon [22,33] and may have an important role to guide endoscopic investigations, especially if elevated calprotectin levels are found in conjunction with a normal colonoscopy. However, in a number of organic intestinal diseases, e.g. chronic gastritis, small intestinal bacterial overgrowth and celiac disease, fecal calprotectin is not elevated [34-36]. In our study, only patients with mucosal breaks were classified as significant findings.

Using faecal calprotectin testing in conjunction with EPAGE criteria scores led to a significant reclassification of patients being correctly classified as at higher or lower risk of endoscopic findings than did assessment with appropriateness criteria alone. The use of faecal calprotectin testing as a continuous rather than a categorical parameter (below or above cut-off of 50 ug/g) allowed for a detailed risk stratification of individual patients presenting with abdominal complaints. We estimated the risk for endoscopic findings to be <30% in patients with inappropriate indications for endoscopy. Accordingly, in patients that would require endoscopy according to the risk prediction, diagnostic yield increased compared to the individual use of EPAGE scoring or faecal calprotectin values, especially for colonoscopy.

The clinical implications of our study merit consideration. The combined use of EPAGE scoring and faecal calprotectin testing led to a superior risk stratification in patients with abdominal complaints and increased diagnostic yield in patients requiring endoscopy. This would especially be useful in open-access health care systems, when patients are referred for endoscopy by non-gastroenterologists. The use of an objective, easy-to-use and easy-to-interpret biomarker would decrease the rate of, often unintentionally, inappropriate endoscopies, and would facilitate the decision to perform endoscopy when the indication is driven by the patient’s (or the doctor’s) fear of missing a diagnosis rather than by rational clinical judgment. Accordingly, our data indicate that faecal calprotectin testing should be included in the current EPAGE algorithms or other algorithms devoted to clinical decision-making as to when to perform digestive endoscopy. However, it remains to be determined at what stage of the decision tree the diagnostic impact of faecal calprotectin testing would be greatest. Additionally, future studies should also aim to investigate the cost-effectiveness of appropriateness guidelines, especially when costs of additional biomarkers are taken into account.

There are several potential limitations of the current study. First, this was a post-hoc analysis of a prospective study and EPAGE criteria were applied retrospectively. It has been shown that retrospective analysis of appropriateness criteria is feasible but is found to be imprecise when adjudicating patients to certain indication groups [37]. Second, we carefully assessed the appropriateness of endoscopies using a predefined list of indications and we meticulously reviewed all available data of each individual patient to include only those with sufficient clinical and laboratory information. Of 575 patients originally enrolled, only 51.8% were available for analysis, thus making the analysis susceptible to selection bias. However, when baseline characteristics of patients excluded were compared, patients in the study population were younger and had received more EGDs and fewer colonoscopies but the prevalence of significant endoscopic findings was similar (Additional file 1: Table S1). Third, EPAGE appropriateness guidelines are panel-based opinions and thus present an inherent limitation. Guidelines should be regarded as recommendations but cannot fully replace clinical judgment, especially at an individual level. However, clinical judgment alone is unreliable in predicting organic intestinal disease and hence is an insufficient indicator for assessing appropriateness of GI endoscopy. In addition, gastroenterologists overestimate the appropriateness of their investigations [38]. Fourth, because predicted risks for significant endoscopic findings in inappropriate, uncertain and appropriate endoscopy according to EPAGE criteria are unknown, the reclassification of patients in this study was based on estimated risk-groups. The estimations were based on the available data on diagnostic yield found in the literature of patients with inappropriate, uncertain and appropriate indications for endoscopy according to EPAGE criteria [7,8,10-14]. Fifth, data derived from a single-centre study always need to be replicated in larger, multi-centre studies.

## Conclusion

In conclusion, our study showed that measurement of faecal calprotectin is useful for identifying clinically-significant endoscopic findings in patients with abdominal complaints and when combined with appropriateness guidelines improves the limited diagnostic yield of EPAGE criteria. Larger, prospective studies should investigate if the use of faecal calprotectin testing, possibly in combination with other biomarkers, would warrant inclusion in diagnostic algorithms such as EPAGE guidelines.

## Competing interests

All authors disclose no conflict of interest.

## Authors’ contributions

EB, CB and FSL participated in study concept and design, acquisition of data, analysis and interpretation of data, drafting of the manuscript, and critical revision of the manuscript for significant intellectual content. They also had full access to all of the data in the study and take responsibility for the integrity of the data and the accuracy of the data analysis. MM, PS, FF and LR participated in acquisition of data and critical revision of the manuscript for significant intellectual content. All authors read and approved the final manuscript.

## Pre-publication history

The pre-publication history for this paper can be accessed here:

http://www.biomedcentral.com/1471-230X/14/57/prepub

## Supplementary Material

Additional file 1: Table S1 Patient characteristics of the study population and patients not included in the study. Data are presented as numbers (%). Miscellaneous indications in the study population included diverticulitis (N = 2), NSAID-induced colitis (N = 2) and unspecified proctitis (N = 2). Miscellaneous indications for patients not included in the analysis included diverticulitis (N = 11) and 1 patient with pill-induced esophageal ulcer. Click here for file
