# Supplementary material for: Diagnostic yield of endoscopy in patients with abdominal complaints: incremental value of faecal calprotectin on guidelines of appropriateness
Source: BMC Gastroenterol. 2014 Mar 29;14:57. doi: 10.1186/1471-230X-14-57 (PMC4021405; doi:10.1186/1471-230X-14-57)
Supplement: Additional file 1: Table S1 — Patient characteristics of the study population and patients not included in the study. Data are presented as numbers (%). Miscellaneous indications in the study population included diverticulitis (N = 2), NSAID-induced colitis (N = 2) and unspecified proctitis (N = 2). Miscellaneous indications for patients not included in the analysis included diverticulitis (N = 11) and 1 patient with pill-induced esophageal ulcer. [file 1471-230X-14-57-S1.doc]

**Additional file 1: Table S1**

|  | **Study population (N = 298)** | **Not included in the analysis (N = 277)** | **P-Value** |
| --- | --- | --- | --- |
| **Demographics** |  |  |  |
| Age, median (IQR) | 58 (46 – 67) | 64 (54 – 73) | <0.001 |
| Age >50 years, N (%) | 188 (63.1) | 220 (79.4) | <0.001 |
| Female, N (%) | 164 (55.0) | 147 (53.1) | 0.732 |
|  |  |  |  |
| **Endoscopy** |  |  |  |
| Colonoscopy, N (%) | 224 (75.2) | 234 (84.8) | 0.006 |
| EGD, N (%) | 149 (50.0) | 87 (31.5) | <0.001 |
| Colonoscopy and EGD, N (%) | 75 (25.2) | 45 (16.3) | 0.012 |
| Clinically significant findings | 110 (36.9) | 113 (40.9) | 0.994 |
| *Reflux esophagitis* | *44 (14.8)* | 13 (4.7) | <0.001 |
| *Erosive gastritis* | *13 (4.4)* | 12 (4.3) | 0.845 |
| *Gastric ulcer* | *4 (1.3)* | 7 (2.5) | 0.461 |
| *Stomach cancer* | *2 (0.7)* | 1 (0.4) | 0.947 |
| *Inflammatory bowel disease* | *9 (3.0)* | 13 (4.7) | 0.403 |
| *Microscopic colitis* | *0 (0.0)* | 5 (1.8) | - |
| *Infectious colitis* | *4 (1.3)* | 5 (1.8) | 0.901 |
| *Hyperplastic polyp* | *0 (0.0)* | 12 (4.3) | - |
| *Adenomatous polyp* | *20 (6.7)* | 29 (10.5) | 0.140 |
| *Colonic cancer* | *10 (3.4)* | 4 (1.4) | 0.227 |
| *Miscellaneous* | *6 (2.0)* | 12 (4.3) | 0.173 |

Data are presented as numbers (%). Miscellaneous indications in the study population included diverticulitis (N = 2), NSAID-induced colitis (N = 2) and unspecified proctitis (N = 2). Miscellaneous indications for patients not included in the analysis included diverticulitis (N = 11) and 1 patient with pill-induced esophageal ulcer.
